# Paraquat Preferentially Induces Apoptosis of Late Stage Effector Lymphocyte and Impairs Memory Immune Response in Mice

**DOI:** 10.3390/ijerph16112060

**Published:** 2019-06-11

**Authors:** Yiming Shao, Yifan Zhao, Tingting Zhu, Fen Zhang, Xiuli Chang, Yubin Zhang, Zhijun Zhou

**Affiliations:** School of Public Health and Key Laboratory of Public Health Safety, MOE, Fudan University, Shanghai 200032, China; 17211020027@fudan.edu.cn (Y.S.); 14301020050@fudan.edu.cn (Y.Z.); 18211020029@fudan.edu.cn (T.Z.); 14301020062@fudan.edu.cn (F.Z.); xlchang@fudan.edu.cn (X.C.)

**Keywords:** paraquat, memory immune response, keyhole limpet hemocyanin, apoptosis

## Abstract

Paraquat (PQ) is a toxic non-selective herbicide. To date, the effect of PQ on memory immune response is still unknown. We investigated the impact of PQ on memory immune response. Adult C57BL/6 mice were subcutaneously injected with 2 mg/kg PQ, 20 mg/kg PQ or vehicle control every three days for two weeks. A single injection of keyhole limpet hemocyanin (KLH) at day four after the initial PQ treatment was used to induce a primary immune response; a second KLH challenge was performed at three months post the first KLH immunization to induce a secondary immune response. In steady state, treatment with 20 mg/kg PQ reduced the level of serum total IgG, but not that of IgM; treatment with 20 mg/kg PQ decreased the number of effector and memory lymphocytes, but not naïve or inactivated lymphocytes. During the primary immune response to KLH, treatment with 20 mg/kg PQ did not influence the proliferation of lymphocytes or expression of co-stimulatory molecules. Instead, treatment with 20 mg/kg PQ increased the apoptosis of lymphocytes at late stage, but not early stage of the primary immune response. During the secondary immune response to KLH, treatment with 20 mg/kg PQ reduced the serum anti-KLH IgG and KLH-responsive CD4 T cells and B cells. Moreover, effector or activated lymphocytes were more sensitive to PQ-induced apoptosis in vitro. Treatment with 2 mg/kg PQ did not impact memory immune response to KLH. Thus, treatment with 20 mg/kg PQ increased apoptosis of late stage effector cells to yield less memory cells and thereafter impair memory immune response, providing a novel understanding of the immunotoxicity of PQ.

## 1. Introduction

Paraquat (PQ) is a highly toxic non-selective herbicide that is widely used throughout the world. PQ is known to causes damage to numerous organs including the lung, brain, kidney, heart, liver, and immune system [1,2,3]. Thus, the toxicities of PQ have been broadly investigated [1].

Although the immunotoxicities of PQ are complex, PQ is a confirmed toxicant to both innate and adaptive immune system [3]. For instance, PQ impaired cytotoxic capacity of nature killer (NK) cells via induction of metallothionein in mice [4]. PQ toxicities to the lung and kidney included an increased level of pro-inflammatory cytokines such as interleukin 1β (IL-1β), IL-6 and tumor necrosis factor α (TNFα) [5,6,7,8,9], indicating that innate immune cells such as macrophages and neutrophils were activated by PQ exposure. In addition, PQ was reported to cause oxidative stress both in vivo and in vitro in that reactive oxygen species (ROS) and malondialdehyde (MDA), a product of lipid peroxidation, were increased during PQ exposure [1,10,11,12,13]. Likewise, PQ was also reported to influence adaptive immune response. For instance, although both increased and decreased levels of serum immunoglobulin (Ig) were reported, most studies suggest that PQ might have a suppressive effect on Ig production [14,15,16,17]. Similarly, numerous studies indicated that PQ increased the number of lymphocytes, while others reported that PQ reduced the number of lymphocytes [14,18,19]. Thus, although some studies suggest that PQ might suppress cellular and humoral immunity, it seems that a couple of factors including the dose of PQ, strains and time and routines of PQ exposure may significantly influence the effects of PQ on adaptive immunity.

During a primary immune response to a CD4 T cell-dependent antigen, CD4 T cells become activated, and thereafter they proliferate and differentiate to generate more effector CD4 T cells [20]. During the primary immune response, portions of effector CD4 T cells will be eliminated via apoptosis to avoid autoimmunity, while portions of activated CD4 T cells will become memory CD4 T cells that are more efficient in activation after encountering identical antigens in the future [21]. Thus, it is suggested that memory lymphocytes might be generated at the late stage of a primary immune response [20]. Similar process also occurs in vivo for induction of memory B cells in response to antigens; CD4 T cells, in particular CD4 T follicular cells play an essential role in this process [22]. Obviously, induction of memory immune responses is crucial for host defense against pathogens [23].

Although a variety of studies have been performed to investigate the effects of PQ on immunity [4,14,15,16,17,18,19], to date, the influence of PQ on memory immune response remains incompletely understood. Previously, we performed numerous studies to elucidate the neurotoxicity and lung toxicity of PQ [11,24,25,26,27,28]. In this study, we hypothesized that PQ might impair memory immune response. We performed a group of experiments that directly tested the memory immune responses to the antigen keyhole limpet hemocyanin (KLH) during PQ exposure. We found that PQ impaired memory immune response to KLH likely due to increased apoptosis of effector CD4 T cells and activated B cells at the end of the primary immune response, which is a previously unknown toxicity of PQ to the immune system.

## 2. Materials and Methods

### 2.1. Animals, PQ Treatments and KLH Immunization

Wild-type C57BL/6 (B6) mice at 6 to 8 weeks (wks) of age were purchased from Shanghai SLAC Laboratory Animal Co Ltd., China. Males and females were equally used. Mice were housed in a specific pathogen free animal facility with an artificial 12-hour (hr) light on/off cycle, a constant temperature (23 °C) and humidity (30%) at Fudan University. 6 mice were housed per cage with free access to water and food ad libitum; males and females were housed separately.

PQ was purchased from Sigma (St Louis, MO, USA). Mice were subcutaneously injected with 2 mg of PQ per mouse body weight (mg/kg), 20 mg/kg or PBS as vehicle control every three days for two weeks. At day 4 post the initial PQ treatment, portions of mice received a subcutaneous injection of KLH (5 mg/kg, Sigma) or complete Freund adjuvant (CFA, Sigma) as vehicle control for induction of a primary immune response. At 3 months post the last PQ injection, mice received a second KLH (5 mg/kg) or CFA to induce a memory immune response.

The doses of PQ and KLH used for this study were bases on the previously published literatures [29,30,31]. During the whole period of our study, no differences of general appearance, body weight, and water and food intake were observed by PQ exposure.

This study was approved by the ethics committee of animal use at Fudan University. All animals were operated at a minimal level of suffering during the whole period of study.

### 2.2. Flow Cytometry and Antibodies

Single cell suspensions were prepared from mesenteric lymph nodes (LN) and spleens after erythrocytes were lysized with an ammonia-based buffer. Briefly, we placed the LN and spleens in Petri dishes containing ice-cold PBS and then used 2 glass slides to rub the organs to make single cell suspensions. The single cell suspensions were spun down and the supernatants were discarded. After that, 1 mL of erythrocytes lysis buffer comprised of 8 g/L NH_4_Cl and 100 mg/L KH_2_PO_4_ was added into each sample. The cells were mixed thoroughly and then incubated at room temperature (RT) for 5 minutes (min). After that, 10 mL of PBS was added to each sample and gently mixed. The cells were spun down and supernatants were discarded. The cells were re-suspended in PBS containing 5% fetal bovine serum (FBS) (4 ml for each sample). Then the cell concentrations were counted with an automatic cell counter (Nexcelon). The total number of cells of LN and spleens were comparable between PQ-treated mice and control mice. Antibodies included CD44-PB (1M7), CD62L-PerCP (MEL-14); CD3-FITC (17A2), CD3-PB (17A2), CD4-APC (GK1.5), CD19-FITC (6D5), CD19-PE-Cy7 (6D5), CD80-PE (16-10A1); CD40-PE (3/23), ICOS-PE (7E.17G9), CD154-PE (MR1), OX40-PE (OX-86) and 7AAD (Biolegend, San Diego, CA); I-A-FITC (G155-178), rat IgG isotype-FITC, unconjugated anti-CD16/32 (2.4G2) (Fc block, BD Biosciences, San Jose, CA); Ki67-FITC (SolA15), Ki67-PE (SolA15), Annexin V-FITC (VAA-33) and active caspase-3 staining kit (Invitrogen, Waltham, MA). We placed 2 × 10^6^ cells/sample for each staining. Fc block was used to incubate with cells on ice for 20 min prior to surface staining. Surface staining was performed by incubating cells with antibodies on ice for 30 min in dark. Annexin V staining was performed by incubating cells with anti-Annexin V antibodies at RT in dark for 20 min in the presence of Annexin V binding buffer (Biolegend) after surface staining. Intracellular cleaved caspase-3 staining and nuclear Ki67 staining were performed after surface staining based on the protocols provided by the kits for intracellular staining and nuclear staining, respectively, which were purchased from BD Biosciences. Ki67^+^ cells were gated based on Fluorescence Minus One (FMO) controls. Cells were analyzed with an LSRFortessa instrument (BD Biosciences) and data were analyzed with a FlowJo software (FlowJo LLC, Ashland, OR, USA).

### 2.3. Enzyme-Linked Immunosorbent Assay (ELISA)

Mouse IgM and IgG ELISA kits were purchased from Biolegend to quantify serum IgM and IgG, respectively. Protocols were according to the instructions provided by the manufacturer. Briefly, goat anti-mouse IgM μ chain specific antibodies or goat anti-mouse IgG γ chain specific antibodies (1 μg/100 μL coating buffer/well) were coated in ELISA plates as capture antibodies. Then the unconjugated capture antibodies were discarded and thereafter the plates were blocked with PBS containing 5% bovine serum albumin (BSA). After that, mouse serum (1:500,000 dilution) or standard mouse IgM or IgG were placed into each well in the plates and the plates were then incubated at RT for 2 hr. Horseradish peroxidase (HRP)-conjugated goat anti-mouse Ig whole molecule antibodies were used as detection antibodies. 3,3′,5,5′-tetramethylbenzidine was used as substrate and 1 M sulphuric acid was used to stop the HRP-substrate reaction.

For detecting serum anti-KLH IgG, ELISA plate was coated with KLH (1 μg/100 μL in 0.1 M sodium bicarbonate buffer; Sigma). After blocking with 5% fish gelatin in PBS (Sigma), mouse serum (1:100 dilution) was used as primary antibodies. HRP-conjugated goat anti-mouse IgG Fc specific antibodies (Sigma) were used as detection antibodies. 3,3′,5,5′-tetramethylbenzidine (Sigma) was used as substrate and 1 M sulphuric acid was used to stop the HRP-substrate reaction.

The plates were read for optical density (OD) 450 through a micro-plate reader (BioTek, Winooski, VT, USA).

### 2.4. In Vitro KLH Stimulation 

Mice that had received the 1st KLH immunization or CFA were used for in vitro KLH stimulation assay. Splenic cells were harvested at 3 months post the last PQ injection prior to the second KLH injection in vivo. The splenic cells were cultured in vitro in 96-well plates with complete Dulbecco Modified Eagle Medium (DMEM) containing 20% FBS (Gibco) (10^6^ cells/200 μL/well) in the presence of KLH (25 μg/mL) or CFA as vehicle control. After culture for 48 hr at 37 °C and 5% CO_2_, the cells were evaluated for proliferative CD4 T cells and B cells by detecting their nuclear expression of Ki67 [32].

### 2.5. In Vitro PQ Treatment

Naïve CD4 T cells (CD3^+^CD4^+^CD44^−^CD62L^+^), effector CD4 T cells (CD3^+^CD4^+^CD44^+^CD62L^−^), memory CD4 T cells (CD3^+^CD4^+^CD44^+^CD62L^+^), I-A^low^ B cells (I-A^low^CD19^+^CD80^−^), I-A^hi^ B cells (I-A^low^CD19^+^CD80^−^) and memory B cells (CD19^+^CD80^−^) were purified from spleens of regular B6 mice via a fluorescence-activated cell sorting (FACS) instrument (Aria^TM^ II, BD Biosciences). The purified subtypes of lymphocytes were cultured in vitro in 96-well plates with complete DMEM containing 20% FBS (Gibco) (10^5^ cells/200 μL/well) in the presence or absence of PQ (30 μM, 150 μM or vehicle control). After culture for 24 hr at 37 °C and 5% CO_2_, the cells were evaluated for apoptosis by detecting their surface expression of Annexin V in live cells (7AAD^−^).

### 2.6. Statistics

Data were presented as mean ± standard deviation. Because no sex differences were observed, data from males and females were pooled for analyses. Data were first compared using one-way ANOVA. If there was a significant difference, pos-hoc Student–Newman–Keuls test was used to compare different groups. *p* < 0.05 was used as the level for a significant difference.

## 3. Results

### 3.1. PQ Reduced the Level of Serum IgG and Selectively Decreased the Number of Effector and Memory Lymphocytes during Steady State

**Figure 1:**
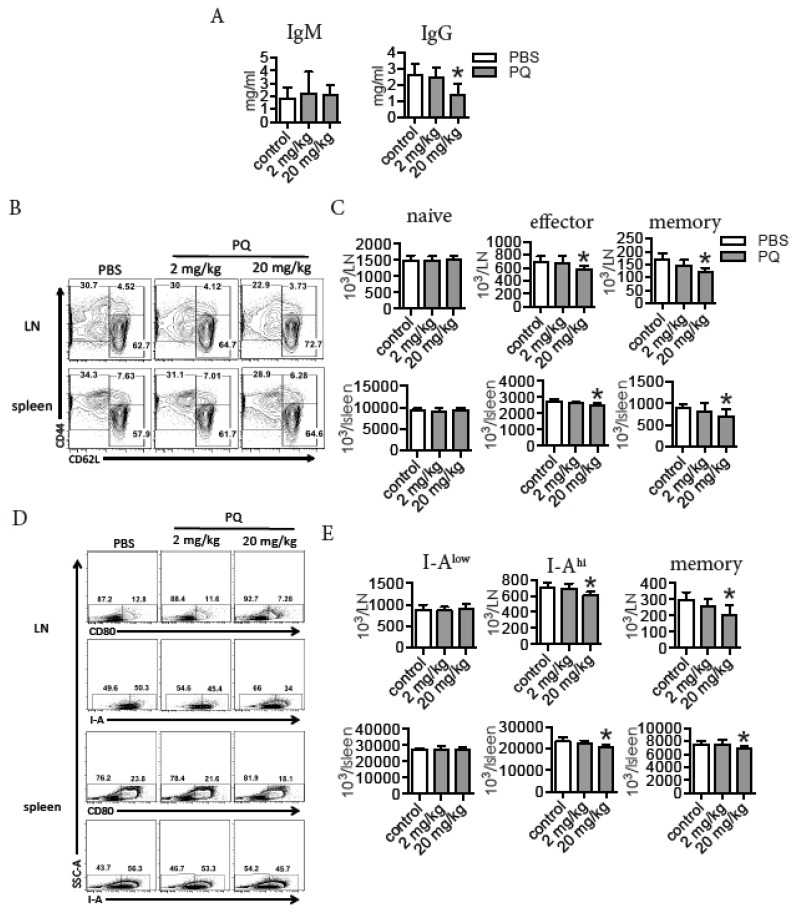
Paraquat (PQ) reduced the level of serum IgG and selectively decreased the number of effector and memory lymphocytes during steady state. B6 mice were treated with PQ for 2 wks and thereafter serum antibodies and CD4 T cells and B cells in the LN and spleen were measured. (**A**) Serum IgM and serum IgG. (**B**) Representative flow plots for naïve (CD62L^+^CD44^−^), effector (CD62L^−^CD44^+^) and memory (CD62L^+^CD44^+^) CD4 T cells in the LN and spleen; CD4 T cells were gated on CD3^+^CD4^+^ cells. (**C**) Quantification of naïve, effector and memory CD4 in the LN and spleen as indicated in B. (**D**) Representative flow plots for I-A^low^ (CD80^−^I-A^low^), I-A^hi^ (CD80^−^I-A^hi^) and memory (CD80^+^) B cells in the LN and spleen; B cells were gated on CD19^+^ cells. (**E**) Quantification of I-A^low^, I-A^hi^ and memory B cells in LN and spleen as indicated in D. A total of 6 to 7 mice were used for each group. Asterisk indicates a significant difference compared to the counterpart control group.

To investigate the effects of PQ on memory immune response, we first quantified serum IgM and IgG in mice after exposure to PQ for 2 wks. Treatment with 20 mg/kg PQ reduced the level of serum IgG, but not that of serum IgM (Figure 1A). Further analyses indicated that treatment with 20 mg/kg PQ reduced the number of effector CD4 T cells (CD3^+^CD4^+^CD44^+^CD62L^−^) and memory CD4 T cells (CD3^+^CD4^+^CD44^+^CD62L^+^), but not that of naïve CD4 T cells (CD3^+^CD4^+^CD44^−^CD62L^+^) in the LN and spleen (Figure 1B). Moreover, treatment with 20 mg/kg PQ decreased the number of activated B cells expressing high level of MHC-II (I-A^hi^) [33] and memory B cells expressing CD80 [32,34], but not that of the less activated B cells expressing low level of MHC-II (I-A^low^) [33] in the LN and spleen (Figure 1C). Thus, treatment with 20 mg/kg PQ could potentially impair memory immune response.

### 3.2. PQ Impaired Memory Immune Response to KLH 

**Figure 2:**
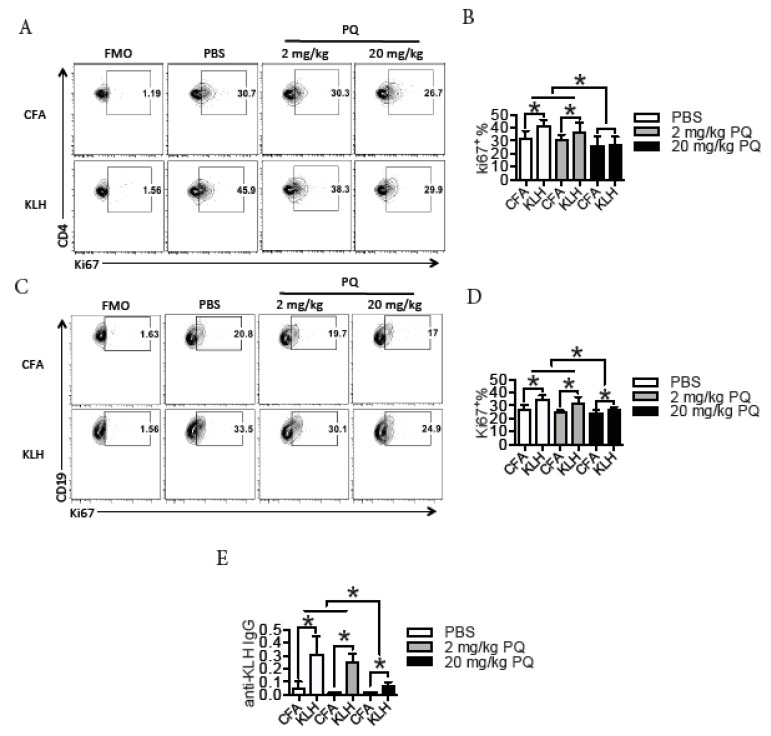
PQ impaired memory immune response to keyhole limpet hemocyanin (KLH). B6 mice treated with PQ for 2 wks were immunized with KLH or vehicle control at day 4 post the initial PQ treatment. After 3 months, splenic cells were harvested from portions of mice to culture with KLH in vitro (25 μg/mL) or CFA for 2 days and thereafter proliferative CD4 T cells and B cells were measured (**A**–**D**); portions of mice were challenged with the 2nd KLH treatment or CFA and sera were harvested for anti-KLH IgG detection at day 3 post the 2nd KLH treatment (**E**). (**A**) Representative flow plots for splenic CD4 T cell proliferation (Ki67^+^) after stimulation with KLH in vitro; CD4 T cells were gated on CD3^+^CD4^+^ cells. (**B**) Quantification of proliferative CD4 T cells as indicated in B. (**C**) Representative flow plots for splenic B cell proliferation (Ki67^+^) after stimulation with KLH in vitro; B cells were gated on CD19^+^ cells. (**D**) Quantification of proliferative B cells as indicated in C. (**E**) Serum anti-KLH IgG in PQ-treated mice after the 2nd KLH stimulation. A total of 6 to 8 mice were used for each group. Asterisk represents a significant difference as indicated.

To test whether PQ impaired memory response during an antigen challenge, we immunized the PQ-treated mice with KLH, a known CD4 T cell-dependent antigen that has been broadly used to induce an adaptive immune response [31,35]. At 3 months post the last PQ injection without the second in vivo KLH challenge, splenic cells were isolated and then cultured in vitro in the presence of KLH to test the proliferation of KLH-responsive CD4 T cells and B cells. As predicted, compared to the control mice, mice treated with 20 mg/kg PQ had less KLH-responsive CD4 T cells during KLH challenge in vitro (Figure 2A,B). Accordingly, mice treatment with 20 mg/kg PQ also had less KLH-responsive B cells during KLH challenge in vitro (Figure 2C,D). To directly test the effect of PQ on the memory immune response to KLH, we challenged the PQ-treated mice that had received the first KLH immunization with a second KLH treatment, and thereafter measured serum KLH-specific IgG production. As shown in Figure 2E, treatment with 20 mg/kg PQ significantly reduced the level of serum anti-KLH IgG during the memory immune response to KLH. Collectively, these experiments demonstrated that PQ could impair the memory immune response to antigens.

### 3.3. PQ Did Not Influence the Proliferation of CD4 T Cells and B Cells during the Primary Immune Response to KLH

Upon stimulation by a CD4 T cell-dependent antigen during a primary immune response, CD4 T cells and B cells become activated and proliferate to generate more cells [20,22]. We next asked whether the reduced number of effector and memory lymphocytes by PQ was due to suppressed proliferation at early stage of activation. To test this, we measured proliferation of CD4 T cells and B cells in LN and spleen at day 4 post the 1st KLH immunization during PQ exposure, based on their nuclear expression of Ki67 as we previously reported [32,36]. We found that KLH induced CD4 T cell proliferation in LN and spleen; PQ did not influence the proliferation of CD4 T cells stimulated by KLH (Table 1). Likewise, KLH also drove the proliferation of B cells in the LN and spleen, and PQ treatment did not influence this process (Table 1). We therefore concluded that PQ did not affect the proliferation of lymphocytes during the primary immune response.

### 3.4. PQ Did Not Influence the Expression of Co-Stimulatory Molecules between CD4 T Cells and Antigen Presenting Cells (APC) during the Primary Immune Response to KLH

Full activation of CD4 T cells requires the interaction of numerous co-stimulatory molecules between CD4 T cells and APC, which may influence the formation of memory immune response [37]. We therefore measured the expression multiple co-stimulatory molecules on CD4 T cells and APC in the LN and spleen after primary response to KLH during PQ exposure. At day 4 post KLH immunization, CD4 T cells had increased surface expression of OX40, ICOS and CD154 in the LN and spleen, and PQ did not affect their expression (Table 1). In addition, mice treatment with PQ or vehicle control had identical increased surface expression of MHC II and CD40 in B cells in the LN and spleen after KLH immunization (Table 1). Mice treated with PQ or vehicle control also had comparable increased surface expression of MHC II, CD80 and CD40 in DC in the LN and spleen during primary immune response to KLH (Table 1). These data indicate that PQ impairment on memory immune response was not due to the altered expression of co-stimulatory molecules in immunological synapse.

### 3.5. PQ Induced Apoptosis of CD4 T Cells and B Cells at Early Stage, but not at Late Stage of the Primary Immune Response to KLH

**Figure 3:**
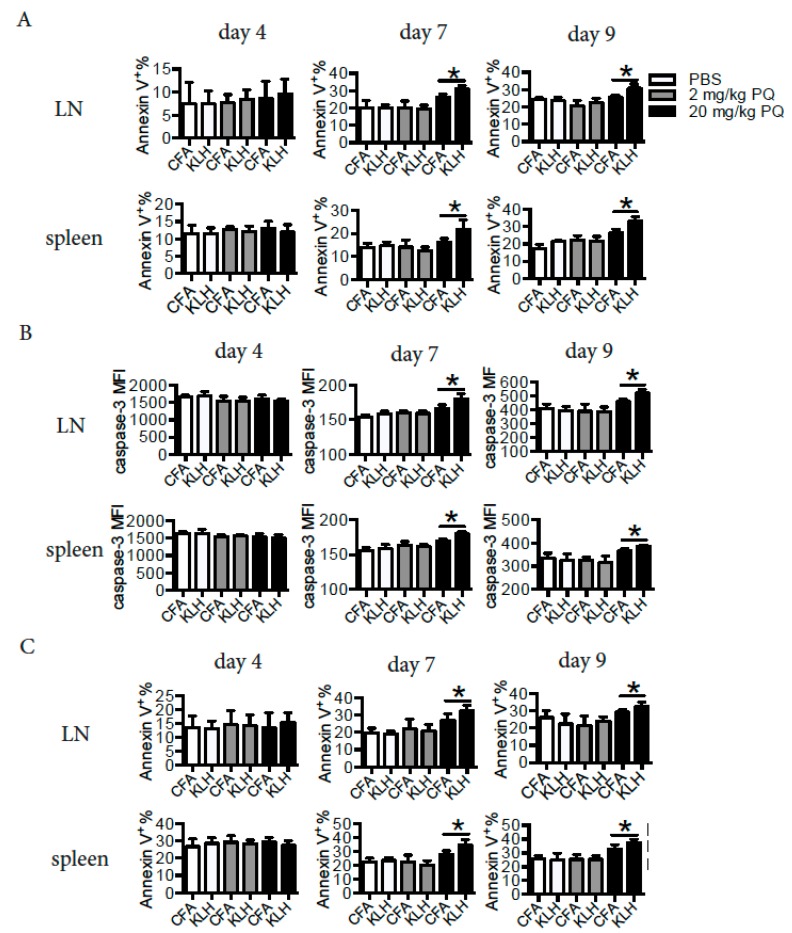
PQ induced apoptosis of CD4 T cells or B cells at late stage, but not at early stage during the primary immune response to KLH. B6 mice treated with PQ for 2 wks were immunized with KLH at day 4 post the initial PQ treatment. Apoptosis of CD4 T cells (CD3^+^CD4^+^) and B cells (CD19^+^) in the LN and spleen were evaluated at day 4, 7, and 9 post the KLH immunization, respectively. (**A**) Surface expression of Annexin V on CD4 T cells. (**B**) Intracellular cleaved (active) caspase-3 expression in CD4 T cells. (**C**) Surface expression of Annexin V on B cells. A total of 6 mice were used for each group. Asterisk represents a significant difference between groups as indicated.

During the primary immune response, naïve CD4 T cells and B cells become activated and thereafter differentiate into effector cells, which eventually may die or give rise to memory cells [20,22]. We asked whether the reduced number of memory cells by PQ was associated with increased apoptosis of activated cells during a primary immune response. To test this hypothesis, we measured the apoptosis of CD4 T cells and B cells at different time points during the primary immune response to KLH. Interestingly, treatment with PQ did not influence the apoptosis of CD4 T cells at day 4 post the KLH immunization, while treatment with 20 mg/kg PQ increased the apoptosis of CD4 T cells at day 7 and day 9 post the KLH immunization in the LN and spleen, based on their surface expression of Annexin V and cytosolic active (cleaved) caspase-3 (Figure 3A,B). Consistently, treatment with 20 mg/kg PQ increased the apoptosis of B cells in the LN and spleen at day 7 and day 9, but not day 4 post KLH immunization, based on their surface expression of Annexin V (Figure 3C). Hence it is likely that PQ selectively drove late stage effector cells, but not early stage of effector cells, to die during a primary immune response.

### 3.6. PQ Preferentially Promoted the Apoptosis of Effector CD4 T Cells and Activated B Cells

**Figure 4:**
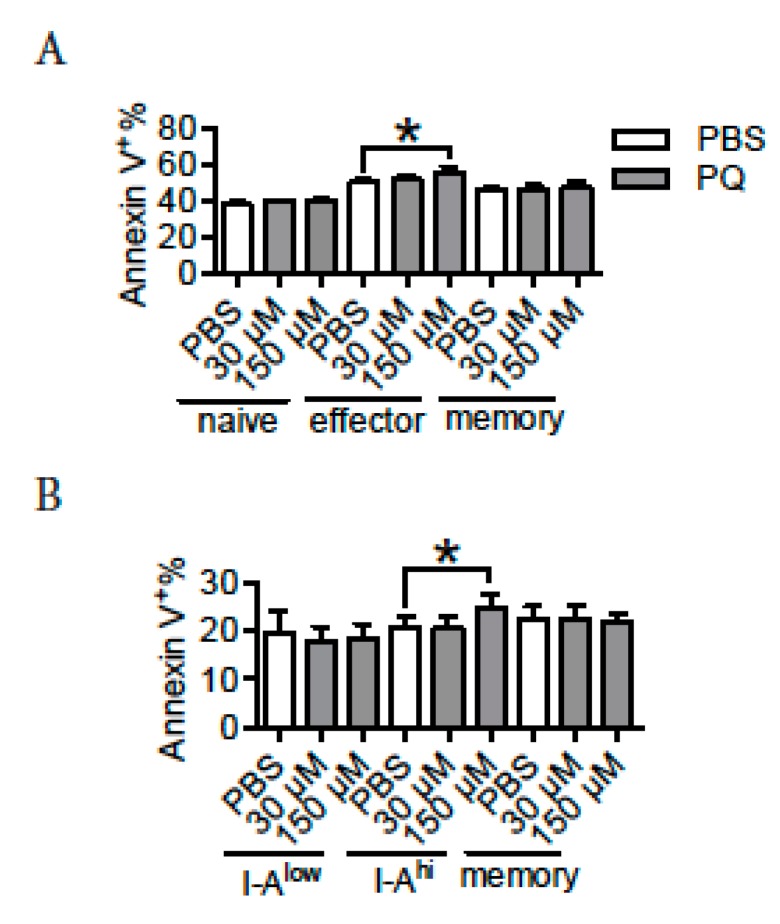
PQ preferentially induced apoptosis in effector lymphocytes relative to naïve or memory lymphocytes. Different subtypes of splenic CD4 T cells (naïve: CD3^+^CD4^+^CD62L^+^CD44^−^; effector: CD3^+^CD4^+^CD62L^−^CD44^+^; memory: CD3^+^CD4^+^CD62L^+^CD44^+^) and B cells (I-A^low^: CD19^+^I-A^low^CD80^−^; I-A^hi^: CD19^+^I-A^hi^CD80^−^; memory: CD19^+^CD80^+^) from regular B6 mice were purified and thereafter cultured in vitro in the presence or absence of PQ. After 24 hr, apoptosis evaluation by surface expression of Annexin V was performed. (**A**): Surface expression of Annexin V on naïve, effector and memory CD4 T cells after PQ treatment in vitro. (**B**): Surface expression of Annexin V on I-A^low^, I-A^hi^ and memory B cells after PQ treatment in vitro. A total of 12 samples were used for each group. Asterisk represents a significant difference between groups as indicated.

PQ selectively drove the apoptosis of CD4 T cells and B cells at late stage of an immune response, indicating that multiple subtypes of lymphocytes may differ in response to PQ-induced apoptosis. To test this, naive CD4 T cells (CD3^+^CD4^+^CD44^−^CD62L^+^), effector CD4 T cells (CD3^+^CD4^+^CD44^+^CD62L^−^), memory CD4 T cells (CD3^+^CD4^+^CD44^+^CD62L^+^), I-A^low^ B cells (I-A^low^CD19^+^CD80^−^), I-A^hi^ B cells (I-A^hi^CD19^+^CD80^−^) and memory B cells (CD19^+^CD80^+^) were purified from spleen and thereafter cultured in vitro in the presence or absence of PQ to evaluate their apoptosis based on their surface expression of Annexin V. We notice that treatment with 150 μM PQ increased the apoptosis of effector CD4 T cells, but not that of naïve or memory CD4 T cells (Figure 4A). Moreover, treatment with 150 μM PQ drove the apoptosis of I-A^hi^ B cells, but not that of I-A^low^ or memory B cells (Figure 4B). Therefore, PQ preferentially drove the apoptosis of effector CD4 T cells and activated B cells relative to naïve and memory cells.

## 4. Discussion

In this study, we revealed that PQ impaired memory immune response to KLH. Specifically, PQ preferentially induced apoptosis of effector or activated CD4 T cells and B cells at late stage during a primary response, possibly causing a reduction of memory cell generation.

Treatment with high dose of PQ (20 mg/kg) selectively reduced the level of serum IgG, but not that of IgM, indicating that PQ might potentially affect memory immune response. The reduction of serum anti-KLH after the 2nd KLH challenge by PQ exposure demonstrated that PQ impaired the memory immunity in response to an antigen. Typically, memory immune cells might be derived from activated or effector cells during the primary immune response [20,22]. The PQ treatment was only performed during the period of primary immune response to KLH, indicating that the impaired memory response observed during the 2nd KLH challenge was due to the event occurring during period of memory immune cell generation.

Multiple factors can influence the formation of memory immune CD4 T cells and B cells [20,22]. During an immune response, CD4 T cells become activated after signaling provided by APC, and thereafter these CD4 T cells proliferate to differentiate into effector cells [20]. Based on the observation that CD4 T cells and B cells had identical proliferation, and CD4 T cells and APC had comparable surface expression of co-stimulatory molecules at the early stage of the primary immune response to KLH, it is likely that PQ did not influence the activation of CD4 T cells. Therefore, it is possible that PQ interfered the events occurring at the late stage of the primary immune response to KLH. At the late stage of a primary immune response, it is possible that the fate of effector cells is to die or become memory cells [20,38]. Interestingly, PQ drove the apoptosis of CD4 T cells and B cells at day 7 and 9, but not at day 4 during the primary immune response to KLH, indicating that PQ preferentially induced the apoptosis of lymphocytes at the late stage, but not at the early stage of an immune response. Thus it is likely that PQ caused more late stage effector CD4 T or activated B cells to die, leading to a decreased proportion of them to differentiate into memory cells. This is also supported by the observation that PQ reduced the number of effector CD4 T cells and activated B cells in steady state and preferentially increased the apoptosis of effector CD4 T cells and activated B cells (I-A^hi^) in vitro.

PQ was reported to drive the apoptosis of various types of somatic cells both in vivo and in vitro [39,40,41]. In the process, dysfunction of mitochondria and induction of ROS were suggested to be critically involved [42]. Thus, it is possible that PQ might preferentially cause apoptosis in cells that are more prone to ROS production. In steady state, immune cells may possess certain level of ROS to maintain their basic physiological activities, and upon stimulation, immune cells may increase the expression of ROS in response to environmental stressors [43]. However, relative to naïve CD4 T cells and memory CD4 T cells, albeit more activated, effector CD4 T cells have a relatively lower level of oxidative state [43]. Thus, PQ-induced apoptosis that preferentially occurred in effector CD4 T cells might be independent of the level of ROS. Interestingly, naïve CD4 T cells preferentially use apoptosis to die in vivo, while activated or effector CD4 T cells preferentially use necroptosis or autophagy to die in vivo [44]. Indeed, PQ was reported to induce autophagy [45]. Thus, it is possible that PQ activated autophagy pathway to drive apoptosis of effector CD4 T cells, which might be considered as an intrinsic pathway for cellular apoptosis. The intrinsic difference of death pathway preferred by naïve and effector CD4 T cells might be the reason for their differential responses to PQ-induced death, although the mechanism remains to be elusive.

PQ is known to cause pulmonary fibrosis, and therefore one limitation of this study was that the lung toxicity in mice after treatment with PQ was not evaluated. In addition, because CFA is an immunomodulator, we did not include a real negative control for the KLH immunization experiment, which was another limitation for this study.

## 5. Conclusions

In conclusion, we found that PQ preferentially induced the apoptosis of late stage effector cells during a primary immune response, possibly resulting in less proportion of them to differentiate into memory cells and the concomitant impaired memory immune response. Our findings may contribute to the current literature on PQ toxicity.

## Figures and Tables

**Table 1:** Assessment of leukocyte co-stimulatory marker expression and lymphocyte proliferation in mice treated with PQ at day 4 post the first KLH immunization.

Cells	Organs Treatments	LN						Spleen					
		PBS		2 mg/kg PQ		20 mg/kg PQ		PBS		2 mg/kg PQ		20 mg/kg PQ	
		CFA	KLH	CFA	KLH	CFA	KLH	CFA	KLH	CFA	KLH	CFA	KLH
CD4 T cells	Ki67 (%)	24.1 ± 5.7	39.3 ± 1.9 *	27.7 ± 3.3	39.2 ± 2.9 *	26.0 ± 3.4	39.0 ± 3.0 *	19.1 ± 2.0	26.3 ± 1.2 *	20.4 ± 1.2	27.2 ± 1.9 *	20.4 ± 1.2	26.8 ± 0.9 *
	OX40 (%)	18.5 ± 1.6	21.8 ± 1.1 *	19.4 ± 0.9	22.5 ± 2.6 *	19.0 ± 0.6	22.2 ± 1.3 *	10.2 ± 0.9	12.2 ± 0.7 *	9.7 ± 1.1	12.4 ± 0.6 *	10.4 ± 1.1	12.3 ± 0.5 *
	ICOS (%)	12.5 ± 1.1	25.3 ± 2.7 *	12.6 ± 1.2	24.3 ± 2.7 *	13.4 ± 1.4	26.2 ± 4.1 *	11.5 ± 0.8	14.7 ± 1.0 *	11.7 ± 0.5	14.4 ± 0.8 *	11.3 ± 1.1	14.6 ± 0.8 *
	CD154 (%)	4.5 ± 0.9	7.5 ± 2.1 *	4.8 ± 0.4	6.9 ± 1.1 *	5.1 ± 0.6	7.5 ± 1.0 *	3.3 ± 0.9	5.3 ± 0.6 *	3.6 ± 0.4	4.8 ± 0.4 *	3.7 ± 0.5	5.1 ± 0.5 *
B cells	Ki67 (%)	13.8 ± 1.1	16.9 ± 0.9 *	13.7 ± 1.5	16.5 ± 0.5 *	13.7 ± 1.1	17.1 ± 0.7 *	20.8 ± 2.4	26.0 ± 1.4 *	21.2 ± 1.5	25.6 ± 1.2 *	21.0 ± 2.0	26.3 ± 1.0 *
	I-A (MFI)	5529 ± 646	8621 ± 1209 *	4899 ± 847	7728 ± 794 *	5683 ± 512	7747 ± 693 *	1263 ± 121	1708 ± 144 *	1151 ± 161	1677 ± 79 *	1106 ± 98	1791 ± 166 *
	CD40 (MFI)	1146 ± 202	1558 ± 144 *	952 ± 136	1598 ± 163 *	1035 ± 175	1519 ± 113 *	978 ± 204	1403 ± 105 *	915 ± 213	1397 ± 150 *	990 ± 169	1365 ± 86 *
DC	I-A (MFI)	7191 ± 681	9705 ± 564 *	7393 ± 519	9357 ± 411 *	6882 ± 521	9051 ± 444 *	1413 ± 157	2024 ± 116 *	1423 ± 130	1968 ± 168 *	1401 ± 138	1947 ± 75 *
	CD80 (MFI)	6507 ± 1065	8816 ± 476 *	6788 ± 918	8418 ± 635 *	6799 ± 625	8900 ± 626 *	4943 ± 337	5654 ± 271 *	4861 ± 364	5768 ± 239 *	5142 ± 258	6058 ± 589 *
	CD40 (MFI)	1142 ± 105	1545 ± 111 *	1030 ± 185	1637 ± 145 *	1061 ± 200	1483 ± 100 *	695 ± 124	1004 ± 157 *	736 ± 109	1125 ± 215 *	754 ± 151	1032 ± 109 *

Note: Data were presented as mean ± STD. A total of 6 mice were used for each group. MFI indicates mean fluorescence intensity. Asterisk indicates a significant difference compared to the counterpart control.

## References

[1] Dinis-Oliveira R.J., Duarte J.A., Sanchez-Navarro A., Remiao F., Bastos M.L., Carvalho F. (2008). Paraquat poisonings: Mechanisms of lung toxicity, clinical features, and treatment. Crit. Rev. Toxicol..

[2] Yamada K., Fukushima T. (1993). Mechanism of cytotoxicity of paraquat. II. Organ specificity of paraquat-stimulated lipid peroxidation in the inner membrane of mitochondria. Exp. Toxicol. Pathol..

[3] Wu Q., Xu Q., Jian X., Wang H., He X., Gao B., Wang K., Kan B. (2018). A new sight for paraquat poisoning from immunology. Immunopharmacol. Immunotoxicol..

[4] Lim J.H., Won J.H., Ahn K.H., Back M.J., Fu Z., Jang J.M., Ha H.C., Jang Y.J., Kim D.K. (2015). Paraquat reduces natural killer cell activity via metallothionein induction. J. Immunotoxicol..

[5] Qian J., Ye Y., Lv L., Zhu C., Ye S. (2014). FTY720 attenuates paraquat-induced lung injury in mice. Int. Immunopharmacol..

[6] Qian J., Liu L., Chen L., Lu X., Zhu C. (2015). Increased toll-like receptor 9 expression is associated with the severity of paraquat-induced lung injury in mice. Hum. Exp. Toxicol..

[7] Harchegani A.L., Hemmati A.A., Nili-Ahmadabadi A., Darabi B., Shabib S. (2017). Cromolyn Sodium Attenuates Paraquat-Induced Lung Injury by Modulation of Proinflammatory Cytokines. Drug Res..

[8] Liu Z., Wang X., Wang Y., Zhao M. (2017). NLRP3 inflammasome activation regulated by NF-kappaB and DAPK contributed to paraquat-induced acute kidney injury. Immunol. Res..

[9] Junbo Z., Yongtao Y., Hongbo L., Fenshuang Z., Ruyun L., Chun’ai Y. (2017). Experimental study of sucralfate intervention for paraquat poisoning in rats. Environ. Toxicol. Pharmacol..

[10] Chen Y.W., Yang Y.T., Hung D.Z., Su C.C., Chen K.L. (2012). Paraquat induces lung alveolar epithelial cell apoptosis via Nrf-2-regulated mitochondrial dysfunction and ER stress. Arch. Toxicol..

[11] Chang X., Lu W., Dou T., Wang X., Lou D., Sun X., Zhou Z. (2013). Paraquat inhibits cell viability via enhanced oxidative stress and apoptosis in human neural progenitor cells. Chem. Biol. Interact..

[12] Toygar M., Aydin I., Agilli M., Aydin F.N., Oztosun M., Gul H., Macit E., Karslioglu Y., Topal T., Uysal B. (2015). The relation between oxidative stress, inflammation, and neopterin in the paraquat-induced lung toxicity. Hum. Exp. Toxicol..

[13] Hu X., Shen H., Wang Y., Zhang L., Zhao M. (2019). Aspirin-triggered resolvin D1 alleviates paraquat-induced acute lung injury in mice. Life Sci..

[14] Riahi B., Rafatpanah H., Mahmoudi M., Memar B., Fakhr A., Tabasi N., Karimi G. (2011). Evaluation of suppressive effects of paraquat on innate immunity in Balb/c mice. J. Immunotoxicol..

[15] Okabe M., Nishimoto S., Sugahara T., Akiyama K., Kakinuma Y. (2010). Oral administration of paraquat perturbs immunoglobulin productivity in mouse. J. Toxicol. Sci..

[16] Ma J., Li Y., Niu D., Li Y., Li X. (2014). Immunological effects of paraquat on common carp, *Cyprinus carpio* L.. Fish Shellfish Immunol..

[17] Ma J., Li Y., Wu M., Zhang C., Che Y., Li W., Li X. (2018). Serum immune responses in common carp (Cyprinus carpio L.) to paraquat exposure: The traditional parameters and circulating microRNAs. Fish Shellfish Immunol..

[18] Riahi B., Rafatpanah H., Mahmoudi M., Memar B., Brook A., Tabasi N., Karimi G. (2010). Immunotoxicity of paraquat after subacute exposure to mice. Food Chem. Toxicol..

[19] Hassuneh M.R., Albini M.A., Talib W.H. (2012). Immunotoxicity induced by acute subtoxic doses of paraquat herbicide: Implication of shifting cytokine gene expression toward T-helper (T(H))-17 phenotype. Chem. Res. Toxicol..

[20] Chang J.T., Wherry E.J., Goldrath A.W. (2014). Molecular regulation of effector and memory T cell differentiation. Nat. Immunol..

[21] Schluns K.S., Lefrancois L. (2003). Cytokine control of memory T-cell development and survival. Nat. Rev. Immunol..

[22] McHeyzer-Williams M., Okitsu S., Wang N., McHeyzer-Williams L. (2011). Molecular programming of B cell memory. Nat. Rev. Immunol..

[23] Netea M.G., Joosten L.A., van der Meer J.W., Kullberg B.J., van de Veerdonk F.L. (2015). Immune defence against Candida fungal infections. Nat. Rev. Immunol..

[24] Chang X., Shao C., Wu Q., Wu Q., Huang M., Zhou Z. (2009). Pyrrolidine dithiocarbamate attenuates paraquat-induced lung injury in rats. J. Biomed. Biotechnol..

[25] Huang M., Lou D., Cai Q., Chang X., Wang X., Zhou Z. (2014). Characterization of paraquat-induced miRNA profiling response in hNPCs undergoing proliferation. Int. J. Mol. Sci..

[26] Dou T., Yan M., Wang X., Lu W., Zhao L., Lou D., Wu C., Chang X., Zhou Z. (2016). Nrf2/ARE Pathway Involved in Oxidative Stress Induced by Paraquat in Human Neural Progenitor Cells. Oxid. Med. Cell. Longev..

[27] Lou D., Wang Q., Huang M., Zhou Z. (2016). Does age matter? Comparison of neurobehavioral effects of paraquat exposure on postnatal and adult C57BL/6 mice. Toxicol. Mech. Methods.

[28] Huang M., Yang H., Zhu L., Li H., Zhou J., Zhou Z. (2016). Inhibition of connective tissue growth factor attenuates paraquat-induced lung fibrosis in a human MRC-5 cell line. Environ. Toxicol..

[29] Ortiz M.S., Forti K.M., Suarez Martinez E.B., Munoz L.G., Husain K., Muniz W.H. (2016). Effects of Antioxidant N-acetylcysteine Against Paraquat-Induced Oxidative Stress in Vital Tissues of Mice. Int. J. Sci. Basic Appl. Res..

[30] Javad-Mousavi S.A., Hemmati A.A., Mehrzadi S., Hosseinzadeh A., Houshmand G., Rashidi Nooshabadi M.R., Mehrabani M., Goudarzi M. (2016). Protective effect of Berberis vulgaris fruit extract against Paraquat-induced pulmonary fibrosis in rats. Biomed. Pharmacother..

[31] Heo Y., Zhang Y., Gao D., Miller V.M., Lawrence D.A. (2011). Aberrant immune responses in a mouse with behavioral disorders. PLoS ONE.

[32] Yang Z., Zhao Y., Li Q., Shao Y., Yu X., Cong W., Jia X., Qu W., Cheng L., Xue P. (2019). Developmental exposure to mercury chloride impairs social behavior in male offspring dependent on genetic background and maternal autoimmune environment. Toxicol. Appl. Pharmacol..

[33] Kil L.P., de Bruijn M.J., van Nimwegen M., Corneth O.B., van Hamburg J.P., Dingjan G.M., Thaiss F., Rimmelzwaan G.F., Elewaut D., Delsing D. (2012). Btk levels set the threshold for B-cell activation and negative selection of autoreactive B cells in mice. Blood.

[34] Bemark M., Hazanov H., Stromberg A., Komban R., Holmqvist J., Koster S., Mattsson J., Sikora P., Mehr R., Lycke N.Y. (2016). Limited clonal relatedness between gut IgA plasma cells and memory B cells after oral immunization. Nat. Commun..

[35] Giesecke C., Meyer T., Durek P., Maul J., Preiss J., Jacobs J.F.M., Thiel A., Radbruch A., Ullrich R., Dorner T. (2018). Simultaneous Presence of Non- and Highly Mutated Keyhole Limpet Hemocyanin (KLH)-Specific Plasmablasts Early after Primary KLH Immunization Suggests Cross-Reactive Memory B Cell Activation. J. Immunol..

[36] Li Q., Yang Z., Zhang P., Zhao Y., Yu X., Xue P., Shao Y., Li Q., Jia X., Zhang Q. (2018). Mercury impact on hematopoietic stem cells is regulated by IFNgamma-dependent bone marrow-resident macrophages in mice. Toxicol. Lett..

[37] Hubo M., Trinschek B., Kryczanowsky F., Tuettenberg A., Steinbrink K., Jonuleit H. (2013). Costimulatory molecules on immunogenic versus tolerogenic human dendritic cells. Front. Immunol..

[38] St John A.L., Rathore A.P.S. (2019). Adaptive immune responses to primary and secondary dengue virus infections. Nat. Rev. Immunol..

[39] Jang Y.J., Won J.H., Back M.J., Fu Z., Jang J.M., Ha H.C., Hong S., Chang M., Kim D.K. (2015). Paraquat Induces Apoptosis through a Mitochondria-Dependent Pathway in RAW264.7 Cells. Biomol. Ther..

[40] Yao J., Zhang J., Tai W., Deng S., Li T., Wu W., Pu L., Fan D., Lei W., Zhang T. (2019). High-Dose Paraquat Induces Human Bronchial 16HBE Cell Death and Aggravates Acute Lung Intoxication in Mice by Regulating Keap1/p65/Nrf2 Signal Pathway. Inflammation.

[41] Hu X., Chen L., Li T., Zhao M. (2019). TLR3 is involved in paraquat-induced acute renal injury. Life Sci..

[42] Blanco-Ayala T., Anderica-Romero A.C., Pedraza-Chaverri J. (2014). New insights into antioxidant strategies against paraquat toxicity. Free Radic. Res..

[43] Franchina D.G., Dostert C., Brenner D. (2018). Reactive Oxygen Species: Involvement in T Cell Signaling and Metabolism. Trends Immunol..

[44] Zhan Y., Carrington E.M., Zhang Y., Heinzel S., Lew A.M. (2017). Life and Death of Activated T Cells: How Are They Different from Naive T Cells?. Front. Immunol..

[45] Gonzalez-Polo R.A., Niso-Santano M., Ortiz-Ortiz M.A., Gomez-Martin A., Moran J.M., Garcia-Rubio L., Francisco-Morcillo J., Zaragoza C., Soler G., Fuentes J.M. (2007). Inhibition of paraquat-induced autophagy accelerates the apoptotic cell death in neuroblastoma SH-SY5Y cells. Toxicol. Sci..

